# Emergent Strategies for Natural Products Delivery

**DOI:** 10.3390/pharmaceutics12121179

**Published:** 2020-12-03

**Authors:** Anna Rita Bilia, Vieri Piazzini, Maria Camilla Bergonzi

**Affiliations:** Department of Chemistry, University of Florence, Via Ugo Schiff 6, Sesto Fiorentino, 50019 Florence, Italy; ar.bilia@unifi.it (A.R.B.); vieri.piazzini@gmail.com (V.P.)

Natural products have a wide range of applications with a profound impact in the medical and healthcare fields. In the last decade, numerous delivery systems based on natural products have been developed to optimize their biopharmaceutical properties, to achieve desirable target characteristics, to reduce doses and side effects, and to achieve therapeutic levels of drugs over an extended period. This is generally related to the scarce water solubility, low lipophilicity, and inappropriate molecular size of natural compounds, which undergo structural instability in a biological milieu, rapid clearance, and a high metabolic rate. Additionally, some molecules are destroyed in gastric juice or suffer a massive pre-systemic metabolism in the liver when administered orally, limiting their clinical use. Reduced bioavailability can be also linked to drug distribution/accumulation in non-targeted tissues and organs that increase the side effects, lowering the therapeutic efficacy and patient compliance. Delivery systems represent favorable tools to increase the bioavailability and activities of natural products.

This Special Issue is dedicated to micro and nanosized drug delivery systems that have successfully been developed. The Special Issue includes ten original papers dealing with the different applications of nanocarriers to natural compounds or extracts.

Bosca et al. presents the preparation of nanocarriers with different composition (lipidic or polymeric) and different structure (vesicular or matricial) to obtain aqueous formulations of unmodified plant-extracted chlorophyll, a strongly hydrophobic compound, known as sonosensitizer agents but with a low selectivity towards cancer tissues. Liposomes, solid lipid nanoparticles and poly(lactic-*co*-glycolic acid (PLGA) nanospheres and nanocapsules were considered. All formulations showed high encapsulation efficiency value, around 98%, and stability, except the PLGA nanocapsules. The nanocarriers have been incubated with human prostatic cancer cells (PC-3) and spheroids (DU-145) to assess the up-take and the influence of the different formulations on the chlorophyll sonodynamic effect. The highest sonodynamic cytotoxicity was obtained with chlorophyll loaded into PLGA nanospheres, showing promising results for future clinical investigations on sonodynamic therapy [1]. 

Aggregated Hydroxyapatite Nanoparticles (HAPs) were proposed as a novel delivery system for with Piperine (Pip). HAPs were coated with gum Arabic (GA) and conjugated with folic acid (FA) on the surface to target colon cancer cells in monolayer and spheroids. Pip was loaded into nanoparticles at different pHs, 7.2 and 9.3. The loading capacity was >20% and the encapsulation efficiency reached 72–81 wt % depending on the presence or absence of the GA coating and FA conjugation. Prolonged release was observed with kinetics dependent on pH, surface modification, and coating. The GA polymeric coating conjugated to FA prolongs the release effect because it hinders Pip release. Pip is more soluble in acidic media (pH 5 or 6.8) and it is released inside the tumor cell upon degradation of the GA coating. The fastest release of Pip in vitro was detected at 24 h at pH 5. As the pH increased, the release decreased. The nanoformulations fully inhibited monolayer HCT116 colon cancer cells compared to Caco2 colon cancer and MCF7 breast cancer cells after 72 h, whereas free Pip had a weaker effect. The nanoformulations functionalized with GA e FA had the lowest cytotoxicity towards normal WI-38 fibroblast cells [2].

Ethosomes^®^ and transfersomes^®^ were investigated for the percutaneous delivery of sulforaphane to be applied for the treatment of skin cancer diseases. Both carriers had mean sizes <300 nm, a PdI close to 0.2 and a zeta potential between −20 and −30 mV. The stability studies demonstrated that the most suitable ultradeformable vesicles to be used as topical carriers of sulforaphane were ethosomes^®^ containing ethanol and phospholipon 90 G. The in vitro percutaneous permeation studies through human stratum corneum and epidermis membranes showed an increase of the percutaneous permeation of sulforaphane. This effect is probably due to the presence of ethanol in the composition of ethosomes^®^, which could promote interaction between carriers and lipids of the stratum corneum. The antiproliferative activity of sulforaphane-loaded ethosomes^®^ was tested on SK-MEL 28 and improved anticancer activity was observed in comparison with the free drug [3].

Vanti et al. prepared nanovesicles containing phosphatidylcholine and the natural saponin escin as bilayer-forming components, named escinosomes. They were developed to deliver berberine chloride, a natural quaternary isoquinoline alkaloid used for various skin conditions. These nanovesicles displayed the best characteristics for skin application, particularly optimal polydispersity (0.17) and deformability, high negative zeta potential value, great encapsulation efficiency (about 67%), high stability, and the best release properties of berberine chloride (about 75% after 24 h). Escinosomes maintain escin properties such as hyaluronidase inhibition activity. Nanovesicle permeation properties through artificial membranes and rabbit ear skin were investigated using skin-PAMPA™ and Franz cells were also evaluated [4]. 

Daidzein was formulated in solid dispersions (SDs) in order to enhance its aqueous solubility and bioavailability. Second-generation solid dispersions (SG) that employ amorphous polymers and the drug can be molecularly dissolved or dispersed, third-generation solid dispersions (TG), where a surfactant is added to the system to increase the drug dissolution, and second- and third-generation pH-modulated solid dispersions (SDs and TG pHM-SDs) were produced via spray drying. Daidzein is in an amorphous state in most of the formulations. SDs increased aqueous solubility and in vitro release rate. The TG-pHM SDs showed the highest capability of solubilizing high doses of Daidzein (>20 mg/mL). The SG-pHM SDs had faster and higher release rates compared with the SDs. The TG-pHM SDs also increased the release rate. The enhanced aqueous solubility of TG-pHM SD was reflected by an increase in oral bioavailability [5].

Loepfe and coworkers proposed the preparation of microparticles for controlled delivery of epigallocatechin-3-gallate (EGCG) in the degenerated intervertebral disc (IVD). EGCG was encapsulated by electrospraying of glutaraldehyde-crosslinked gelatin. The EGCG biological activity was retained after electrospray as demonstrate by gene and protein expression of EGCG targets in IVD cells. After one week in the pro-inflammatory 3D cell cultures, EGCG microparticles strongly inhibited IL-1β-dependent expression of IL-6, IL-8, COX-2, MMP1, MMP3, and MMP13. 

The resulting particles were combined with a thermoresponsive carrier to produce an injectable EGCG delivery system. This carrier was in situ-forming Hyaluronic acid-Poly(*N*-isopropylacrylamide) hydrogel, a slowly biodegradable material. The drug delivery system provides sustained release of EGCG. Subsequently, electrospraying was scaled up using the industrial NANOSPIDER™ for further (pre)clinical development. The particles have homogenous spherical morphologies. The produced EGCG microparticles reduced the expression of inflammatory and catabolic mediators in pro-inflammatory 3D cell cultures obtained from primary IVD cells isolated from human donors undergoing IVD surgeries. Combining the EGCG microparticles with the thermoresponsive carrier showed a trend towards modulating EGCG activity/release [6].

Macelli et al. analyzed the chemical fingerprints of four different *Satureja montana* L., three from growing wild populations and one from commercial source. The essential oils (SEOs) and the non-polar volatile fraction were characterized. The authors also prepared oil in water nanoemulsions composed by SEOs and Tween 20 or Tween 80. The nanoemulsions are stable and homogeneous in size. DLS results evidenced that different surfactant and their different ratios lead to formulations with different sizes, in particular as the surfactant content increases, the dimensions decrease. All samples show negative zeta potential values. Minimum inhibitory and minimum bactericidal concentrations of SEOs were determined towards Gram-positive (*Listeria monocytogenes*, *Staphylococcus aureus*, *Staphylococcus haemolyticus*) and Gram-negative clinical isolates (*Escherichia coli*, *Klebsiella pneumoniae*, *Pseudomonas aeruginosa*, and *Serratia marcescens*). MIC and MBC values ranged from 0.39 to 6.25 mg·mL^−1^. Nanoemulsions was able to preserve and improve antimicrobial activity compared to essential oil alone [7]. 

*Posidonia oceanica* L. is a marine plant endemic of Mediterranean Sea, rich in polyphenols and carbohydrates, and it has been shown to inhibit human cancer cell migration. Chitosan nanoparticles and Soluplus polymeric micelles have been developed to increase the aqueous solubility of the hydroalcholic extract of *P. oceanica* leaves (POE) and increase its inhibitory effect on cancer cell migration. Nanoformulations were chemically and physically defined and characterized. The sizes of nanoformulations were around 250 nm for nanoparticles and 55 nm for polymerc micelles. The encapsulation efficiency values are 10.6% and 85.0% for chitosan nanoparticles and Soluplus polymeric micelles, respectively. Both nanoformulations showed excellent physical and chemical stability during storage, and enhanced the solubility of POE. Finally, the inhibitory effect of both nanoformulations was tested on SH-SY5Y cell migration by wound healing assay and compared to that of unformulated POE. Soluplus polymeric micelles improved the inhibitory effect of extract on cell migration probably due to the high encapsulation efficiency and the prolonged release of the extract [8]. 

The study of Beconcini et al. reported the encapsulation of a natural cherry extract (CE) in nanoparticles based on two different quaternary ammonium chitosan derivatives (Ch-der) or poly(lactic-*co*-glycolic acid, PLGA). The anti-inflammatory effect of either free or encapsulated extract was tested on human umbilical vein endothelial cells (HUVEC). Extract and formulations cytotoxicity and protective effect on lipopolysaccharide (LPS)-stressed HUVEC were also considered. Pro- and anti-inflammatory cytokines released by HUVEC were quantified by enzyme-linked immunosorbent assay. Among all formulations, CE-loaded Ch-der nanoparticles showed the highest in vitro uptake and anti-inflammatory activity. Moreover, all nanoparticles reduced the production of nitric oxide and NLRP3 inflammasome, and had a stronger anti-inflammatory effect than the major corticosteroid dexamethasone. The study demonstrates that natural CE protects endothelial cells from inflammatory stress when encapsulated in nanoparticles based on quaternary ammonium chitosan. The CE beneficial effects were directly related with in vitro internalization of CE-loaded nanoparticles [9].

In the study of Fuior et al. naringenin and hesperetin were formulated into PEGylated lipid nanoemulsions coupled with a VCAM-1 recognizing peptide (LNs), targeted to vascular cell adhesion molecule-1 (VCAM-1), which is expressed on activated endothelial cells (ECs). 

LNs have a hydrodynamic size of about 200 nm, negative zeta potential, an encapsulation efficiency of flavonoids higher than 80%, good in vitro stability and steady release of the loaded molecules. LNs were neither cytotoxic to human ECs line EA.hy926, nor provoked in vitro lysis of murine erythrocytes. Flavonoid-loaded LNs, either non-targeted or targeted to the endothelium, were taken up by the EA.hy926 cells in a dose-dependent manner, but dependent on TNF-α only in the case of endothelium-targeted LNs. Moreover, these nanoparticles exerted a significantly higher percentage of inhibition of monocyte adhesion and transmigration to/through the TNF-α-activated endothelium as compared with non-targeted and free flavanones. LPs can deliver polyphenols to activated ECs and have the functional capacity to reduce monocyte infiltration through activated ECs by reducing the nuclear translocation of NF-kB and the production of MCP-1 chemokine [10].
